# Homogenization of Amorphous Solid Dispersions Prepared by Electrospinning in Low-Dose Tablet Formulation

**DOI:** 10.3390/pharmaceutics10030114

**Published:** 2018-08-02

**Authors:** Gergő Fülöp, Attila Balogh, Balazs Farkas, Attila Farkas, Bence Szabó, Balázs Démuth, Enikő Borbás, Zsombor Kristóf Nagy, György Marosi

**Affiliations:** 1 Gedeon Richter Plc., Formulation R&D, Gyömrői Street 19-21, H-1103 Budapest, Hungary; fulop.gergo89@gmail.com; 2 Department of Organic Chemistry and Technology, Budapest University of Technology and Economics, Budafoki út 8. 3, H-1103 Budapest, Hungary; baloghattila5@gmail.com (A.B.); farkasbalazs09@gmail.com (B.F.); farkas.attila88@gmail.com (A.F.); szabob@oct.bme.hu (B.S.); demuth@oct.bme.hu (B.D.); eniko.borbas@gmail.com (E.B.); gmarosi@mail.bme.hu (G.M.)

**Keywords:** carvedilol, poly (vinylpyrrolidone-*co*-vinyl acetate), high-speed electrospinning, high-shear mixing, homogenization, Raman mapping, sieve analysis

## Abstract

Low-dose tablet formulations were produced with excellent homogeneity based on drug-loaded electrospun fibers prepared by single-needle as well as scaled-up electrospinning (SNES and HSES). Carvedilol (CAR), a BCS II class compound, served as the model drug while poly (vinylpyrrolidone-*co*-vinyl acetate) (PVPVA64) was adopted as the fiber-forming polymer. Scanning electron microscopy (SEM) imaging was used to study the morphology of HSES and SNES samples. Different homogenization techniques were compared to maximize homogeneity: mixing in plastic bags and in a high-shear granulator resulting in low-shear mixing (LSM) and high-shear mixing (HSM). Drug content and homogeneity of the tablets were measured by UV-Vis spectrometry, the results revealed acceptably low-dose fluctuations especially with formulations homogenized with HSM. Sieve analysis was used on the final LSM and HSM powder mixtures in order to elucidate the observed differences between tablet homogeneity. Tablets containing drug-loaded electrospun fibers were also studied by Raman mapping demonstrating evenly distributed CAR within the corpus.

## 1. Introduction

Tablets are generally regarded as the most popular and accepted dosage forms in pharmaceutical technology. Their ease of manufacturing and high patient compliance make them an ideal choice for both the industry and therapeutics [1]. Tablets are available with doses ranging from very low (micrograms) to very high (1–2 g). In such extreme cases, manufacturing becomes more challenging which is especially true for microgram dosed formulations. Handling low quantities of active pharmaceutical ingredients (APIs) can often lead to decreased drug content and high relative standard deviation (RSD) values in the final batches [2]. Proper content uniformity (CU) among others is one of the basic requirements of tablets by Pharmacopeias across the world [3] ensuring high quality and patient safety.

In terms of formulation development, amorphous solid dispersions (ASD) are getting more recognition since enhanced dissolution and bioavailability can be achieved this way [4]. Techniques capable of manufacturing ASDs on the industrial scale, such as spray drying (SD) [5] and hot-melt extrusion (HME) [6] have already produced FDA approved formulations such as Raplixa [7] and NuvaRing [8]. However, SD generally requires high capital investment for the equipment and its maintenance, while it has a low thermal efficiency due to the large volume of hot air circulating in the drying chamber [9]. HME usually operates at even higher temperatures that should be avoided in case of thermosensitive components and it is not applicable for ASD formation in case of APIs of very high melting points [10].

On the other hand, both SD and HME are continuous technologies, which are heavily encouraged by the leading regulatory agencies [11]. Continuous manufacturing has multiple advantages over traditional batch methods, such as huge improvement in productivity, time-efficiency, less energy requirement and a reduced amount of actual waste [12]. As of today, three products have been produced and approved this way in the following chronological order: Orkambi (lumacaftor/ivacaftor) manufactured by Vertex [13], Prezista (darunavir) produced by Janssen [14] and Symdeko (tezacaftor/ivacaftor and ivacaftor) also developed by Vertex [15].

Electrospinning (ES) is a unique method enabling the production of ASDs from a polymer solution or melt [16,17]. Applying ES polymer fibers are formed in the micro and nanometer scale by the creation and elongation of an electrified fluid jet [18]. In comparison with the aforementioned ASD producing technologies, ES operates at ambient conditions resulting in a gentle drying process at minimal costs [19]. The electrospun fibers have an enormous surface area where the API is often molecularly dispersed in a polymer matrix, producing a nano-amorphous solid dispersion (NASD) [20,21,22,23]. ES is also being investigated for various biomedical purposes for its advantages in controlled delivery [24,25,26,27], tissue engineering [28,29], wound dressing, enzyme immobilization and biodrug delivery [30]. 

Regarding scaled-up nanofiber production, the high-speed electrospinning (HSES) technology has the capability to produce approximately half kg of product per hour [31] in accordance with industrial guidelines, while the traditional single-needle electrospinning (SNES) technique can only produce a few grams per hour at best [32,33]. HSES achieves this increased output by combining electrostatic and high-speed rotational jet generation [34,35]. There are also other alternate methods such as pressurized gyration [36], infusion gyration [37] and pressure-coupled infusion gyration [38], that have shown the capability of increased nanofiber output. The latest results and their possible future implications are assembled in a most recent review article [39]. 

The tableting of electrospun nanofibrous materials is a rather new concept so only a few studies have been reported that tackle this field [40,41,42]. However, there is no publication dealing with the homogeneity challenges in tablet formulation. Based on our previous experiences, this can be mainly attributed to the flowability concerns during downstream processing of the ES material. Moreover, ensuring homogeneity, and thus, appropriate CU can be a tall order when very low doses are needed especially in the case of tablets based on electrospun fibers [43]. 

**Figure 1:**
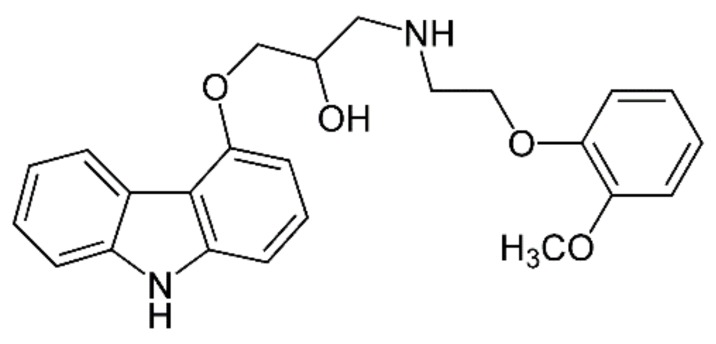
Carvedilol.

To produce ES-based tablets on the industrial scale at any dose, the homogenization characteristics need to be better comprehended and implemented. This work aimed at developing a suitable technology to provide low-dose tablets with proper homogeneity containing electrospun substance prepared by an industrial HSES process. For this purpose, a direct compression (DC) technology was intended to be applied along with industrial tableting by a tablet rotary press. A model system was selected containing carvedilol (CAR, Figure 1), an antihypertensive drug, and PVPVA64, a copolymer as the matrix forming agent (the model drug was selected for analytical reasons, not for any biological relevance at such a low concentration). 

## 2. Materials and Methods

### 2.1. Materials

Carvedilol (CAR) was provided by Sigma-Aldrich (Budapest, Hungary) with purity ≥ 98% and a melting point of 117 °C. Kollidon VA 64 (Copovidone, PVPVA64) was supplied by BASF (Ludwigshafen, Germany), is a vinylpyrrolidone/vinylacetate amorphous copolymer (6:4) with a molecular weight in the range of 45–70 kDa. Lactose monohydrate (Flowlac 100 mesh) was provided by Meggle Pharma (Wasserburg, Germany), while microcrystalline cellulose (Vivapur 112), Croscarmellose sodium (Vivasol) and sodium stearyl fumarate (Pruv) were supplied by JRS Pharma (Rosenberg, Germany).

### 2.2. Single-Needle Electrospinning (SNES)

The single-needle electrospinning experiments were carried out using a spinneret equipped with an NT-35 high voltage DC supply (Unitronik Ltd., Nagykanizsa, Hungary). An electric potential of 25 kV was applied to the spinneret electrode. Sample collection was obtained using a grounded aluminum plate completely covered by aluminum foil. The distance of the spinneret from the collector was set at 15 cm, and the experiments were executed at ambient temperature (25 °C). The electrospinnable solution (Table 1) was dosed utilizing a SEP-10S Plus type syringe pump (Aitecs, Vilnius, Lithuania). The feed rate was 6 mL/h.

### 2.3. High-Speed Electrospinning (HSES)

The scaled-up electrospinning productions were achieved by adopting a high-speed electrostatic spinning setup made up of a stainless steel spinneret with sharp edges and spherical cap geometry controlled by a high-speed motor. The electrospinnable solution was dosed by means of a SEP-10S Plus syringe pump. The flow rate was set at 750 mL/h. A rotational speed of 35,000 rpm was applied to the spinneret, while the voltage was 35 kV through the experiments (NT-65 high voltage DC supply Unitronik Ltd., Nagykanizsa, Hungary). The grounded collector completely covered by aluminum foil was positioned 35 cm from the spinneret in each case. The manufacture of fibers was carried out at ambient temperature (25 °C). More details about HSES can be found in the literature [44].

### 2.4. Low-Shear and High-Shear Homogenization

Homogenizations were carried out in plastic bags (low-shear mixing, LSM) or in a Diosna P-06 high-shear mixer (HSM). The tablet composition is detailed in Table 1.

A 100-mesh amorphous lactose called Flowlac was used as the filler, microcrystalline cellulose type 112 was applied as the binder, croscarmellose sodium (CCS) was adopted as the superdisintegrant and sodium stearyl fumarate (SSF) was implemented as the lubricant. 

In the HSM setup, the chopper blades are set horizontally, which is standard for Diosna high-shear mixers.

In practice, all ingredients except the API and SSF were placed into plastic bags (LSM) or into the HSM, where they were pre-homogenized in 2 min (200 rpm impeller, 600 rpm chopper for HSM). Then the electrospun product or the crystalline CAR was added to the mixture, where it was homogenized in 7 min (200 rpm impeller, 850 rpm chopper for HSM). Upon completion, the mixtures were sieved applying a 500 µm sieve. Finally, SSF was added and blended with the other components in 30 s (200 rpm impeller, 0 rpm chopper for HSM).

### 2.5. Tableting and in Process Control (IPC) Tests

The average tablet weight was set to 100 mg, with the dose of CAR at 50 µg. Tableting was performed on a Riva Piccola tablet rotary press machine using 8 tablet dies with 6 mm diameter and no markings. Table speed was 40 rpm, Fill-O-Matic was 7 rpm and compression force was between 1–1.5 kN. A reference batch was also produced containing crystalline carvedilol as the API. Each batch consisted of 5000 tablets. 

The compressed tablets were evaluated by IPC tests, such as tablet weight, thickness, hardness, friability measurement and time of disintegration. The first 3 were determined by an Erweka MultiCheck apparatus, while friability tests were conducted in a Pharmatest PTFR-A analyzer and disintegration time was measured in a Pharmatest PTZ-Auto disintegration tester. All in all 10 tablets were examined by the MultiCheck, 6 tablets were studied by the PTZ-Auto and tablets with the sum weight of 6.5 g were evaluated by the PTFR-A. Friability tests were made according to the Pharmacopeias with 100 turns in 4 min [45]. 

### 2.6. Scanning Electron Microscopy (SEM) and Fiber Diameter Analysis

Sample morphology was studied by applying a JEOL 6380LVa (JEOL, Tokyo, Japan) type scanning electron microscope. Initially, the samples were secured by virtue of a conductive double-sided carbon adhesive tape, then were coated with an alloy consisting of gold-palladium prior to investigation. The applied accelerating voltage was between 15 and 25 kV, while the working distance was in the range of 12–16 mm. A randomized fiber diameter determination method developed in-house was implemented as described in our previous work [46], *n* = 100 measurements were made on each sample.

### 2.7. Differential Scanning Calorimetry (DSC)

The differential scanning calorimetry (DSC) experiments were made with a DSC 92 Setaram device (Caluire, France). The average sample weight of ~10–15 mg was placed in a closed aluminum pan. A nitrogen purge gas flow of 50 mL/min was used in all experiments. The temperature program started with a preliminary isothermal period that lasted for 1 min at 25 °C. Upon completion, a subsequent linear heating phase ensued from 25 °C to 200 °C at a rate of 10 °C/min. Purified indium standard was applied for calibration.

### 2.8. Raman Mapping

Raman mapping was performed using Horiba Jobin-Yvon LabRAM-type microspectrometer with external 785 nm diode laser source and Olympus BX-40 optical microscope. The laser beam was focused by an objective of 20 × (NA = 0.4) to the tablet surface. The confocal hole of 1000 µm was used in the confocal system. 950 groove/mm grating monochromator dispersed the backscattered light. The spectral range of 460–1680 cm^−1^ was detected as the relevant range with 5 cm^−1^ resolution. Spectral data were collected from 41 × 41 points with 25 µm step size. The acquisition time was 40 s and two spectra were averaged per point. The classical least squares (CLS) method was applied to calculate the concentration of the different materials. All reference spectra (excipients and ES samples) were recorded and the combination of these was used to approximate the spectra of the maps. The so-obtained coefficients were depicted in the maps.

### 2.9. Content Uniformity Analysis

Content uniformity (CU) was measured by UV-Vis spectrometry. Ten random tablets were individually put into 10 mL volumetric flasks where they were disintegrated in an acidic buffer (pH 1) within an ultrasonic bath in 15 min. Upon completion they were filtered by a 0.45 µm polytetrafluoroethylene (PTFE) syringe filter and the concentration of CAR in the acidic buffer (pH 1) was determined by a Hewlett-Packard HP 8453G UV-Vis spectrophotometer (Palo Alto, CA, USA) at a wavelength of 241 nm based on a former calibration. 

### 2.10. Sieve Analysis

Sieve analyses were made on the pure ES material and the final powder mixtures by a Fritsch Analysette 3 Pro Vibratory Sieve Shaker (Idar-Oberstein, Germany). A total weight of 25 g was investigated in each case. The pure ES material and the final powder mixtures were sieved for 10 min with the amplitude set at 0.5. Upon completion, the sieved fractions were weighed and evaluated.

## 3. Results and Discussion

As briefly mentioned at the beginning of this paper, the goal was to achieve highly homogenous NASD containing tablets with low doses of API. By doing so, we could develop a standard method for producing high-quality electrospun products, which meet the requirements of the Pharmacopeias. Fiber morphology of the HSES fibers was studied and compared with SNES samples by SEM. CU was evaluated by UV-Vis spectrometry, while API distribution in the tablets was also studied with Raman mapping and final powder mixture homogeneity was evaluated by sieve analysis.

### 3.1. Electrospinning

The preparation of the ES samples is detailed in Table 2.

Single-needle electrospinning (SNES) is the basic method for preparing NASD; however, its production rate (few grams of product an hour) is way too limited for industrial purposes and applications. Our results fell in line with these assessments since our fiber production with SNES was only 1.8 g per hour. On the other hand, HSES offered a much higher output rate resulting in 225 g of fiber production per hour. In both cases, 96% ethanol was adopted as the solvent since it is accepted by the Pharmacopeia. Using this new technology, it was possible to increase our productivity 125-fold in regard to the dried product. It is encouraging that the output could have been further increased, but in this case, it was only a secondary objective.

### 3.2. Fiber Morphology

**Figure 2:**
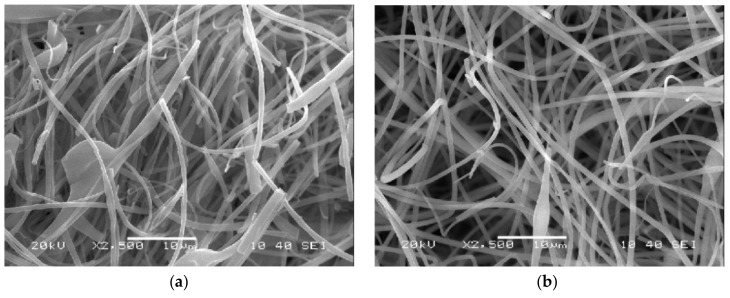
Scanning electron microscopic images of PVPVA64 + 1% CAR fibers prepared by high-speed electrospinning (**a**) and single-needle electrospinning (**b**).

Once the ES materials were obtained their morphology were investigated with SEM. The images are shown in Figure 2. Upon inspecting them the average diameter was 1.02 ± 1.00 µm and 0.80 ± 0.31 µm for the HSES and SNES samples, respectively. However, the HSES sample contained more beads indicating that further research is required to reach optimal morphology. This outcome is likely the result of the fiber-forming technology. No crystalline objects were visible in either case, suggesting their amorphous states.

### 3.3. Differential Scanning Calorimetry

To examine the physical state of carvedilol in the electrospun samples, differential scanning calorimetry measurements were carried out. Physical mixture and pure polymer were used as reference.

**Figure 3:**
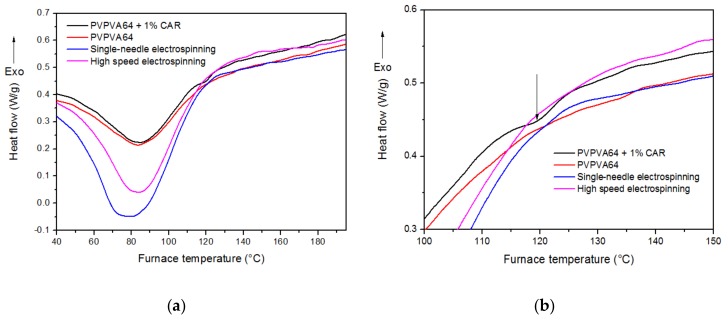
(**a**,**b**) Differential scanning calorimetry (DSC) thermograms of PVPVA64, PVPVA64 + 1% CAR physical mixture and fibers prepared by single-needle and high-speed electrospinning.

The DSC curves are detailed in Figure 3.

According to the thermograms, no endotherm melting peaks of API origin were observable in the electrospun fibers and only a very slight curvature was detected at 120 degrees Celsius in the physical mixture (3.b). Based on these results, we can assume that this faint signal comes from the minimal amount of crystalline carvedilol melting in the aforementioned sample. 

These data further indicate that total amorphization was achieved in both the scaled up (HSES) and regular (SNES) ES samples. The wide endotherm peaks at lower temperatures are related to water evaporation.

### 3.4. Tableting

Once the ES products proved to be amorphous, a direct compression (DC) technology was developed to compress them. DC technology is the safest, easiest and most economical way to produce tablets since it takes the least amount of toll on the ingredients through simple and short homogenization steps [47].

However, in order to successfully apply the technology, proper tableting materials are needed. For our purposes, as they are briefly summarized in Table 1, an amorphous lactose with exceptional flowability called Flowlac was co-processed with microcrystalline cellulose (with low water content < 1.5%). The application of this MCC subtype was preferable since ES products are hygroscopic. The adoption of both materials was desirable since (as it was described in our previous work [48]) the electrospun samples can adhere to their surfaces. Ultimately, this phenomenon sets the foundation to achieve proper homogenization. 

Croscarmellose sodium, specifically developed for manufacturing tablets was used as the superdisintegrant. These agents provide superior disintegration properties compared to regular disintegrants while maintaining a low-dose (1–4%) in the formulation [49]. Lastly, Sodium stearyl fumarate (Pruv), was adopted to lubricate the tablets. It is a hydrophilic agent, that has advantages compared to magnesium stearate such as less sensitivity to over-lubrication [50]. 

For the DC technology only plastic bags, a high-shear mixer, and a tablet rotary press were required. Only the HSES samples were compressed into tablets since the production of sufficient amounts of ingredients by SNES would have taken days to fabricate. Overall, 6 batches were produced 4 of which contained ES product. Two were manually homogenized in plastic bags emulating industrial container homogenization and the other two were homogenized in an HSM. The final two batches contained crystalline carvedilol serving as reference. One was homogenized in LSM conditions and the other by HSM. The batch characteristics are detailed in Table 3.

The HSM suitable for executing multiple technological steps, such as homogenization, granulation, and drying, was expected to work well with the ES product [51]. Once the homogenizations were completed, the mixtures were compressed into tablets using a tablet rotary press without any problems. IPC tests were carried out to evaluate the oral dosage forms. The summary of the results is detailed in Table 3.

According to the IPC results, all batches had roughly the same properties without any unacceptable data. Friability was well below the 1% limit, while disintegration [52] was easily achieved within 30 min both criteria set by the Pharmacopeias. 

### 3.5. Content Uniformity

The measured average API contents and their homogeneity are detailed in Table 4. The batches containing crystalline carvedilol yielded expected results with acceptable API content and high RSD values. These results can be linked to the low quantity of API (250 mg) applied and to the fact that the average particle size of crystalline carvedilol is quite large, that makes its proper homogenization quite difficult in such a small dose. This phenomenon could not be solved even in the HSM since SD and RSD values were still too high, 4.65 and 5.13 respectively. Out of the ES samples, the batches homogenized in HSM proved to be much better than the LSM tablets homogenized in plastic bags under low-shear conditions. To differentiate between the samples, additional measurements were carried out.

### 3.6. Raman Mapping Results

To evaluate the homogeneity differences between LSM and HSM ES tablets, Raman maps were taken of the aforementioned solid dosage forms. Raman mapping is a non-destructive analytical method enabling API homogeneity measurements in pharmaceuticals [53]. Analyses were conducted on individual tablet surfaces, one from each batch. 

**Figure 4:**
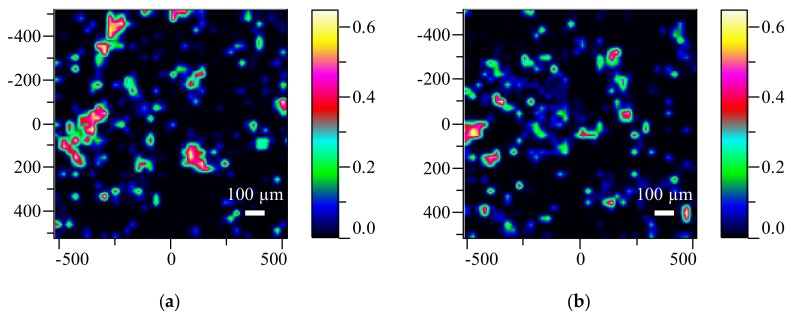
Raman maps of LSM (**a**) and HSM (**b**) ES tablets (colorful images show the distribution of the PVPVA64 polymer).

Since directly searching for the API at such a low-dose (50 µg) with Raman spectrometry is a very challenging task, an indirect method was applied. Given the fact, that the API is molecularly dispersed in the PVPVA64 polymer matrix [54], we can assume that the homogeneity of the API is related to the homogeneity of the polymer. Consequently, the maps were taken accordingly. These results are shown in Figure 4.

Upon inspecting the maps, the distribution of API could be indirectly investigated, but only slight differences were detected within the standalone tablets. This result indicates, that the API containing polymer is evenly distributed in the single solid dosage forms. However, this still does not explain the large inhomogeneity deviations discovered by the CU measurement, which is a result of evaluating multiple tablets at once.

### 3.7. Sieve Analysis

Since the analysis of single tablets did not clarify the deviations between the LSM and HSM, sieve analysis was chosen as an alternative method. A total weight of 25 g (the equivalent of 250 tablets) of LSM and HSM final powder mixtures along with the pure fibers were analyzed, whose results are detailed in Table 5. Both LSM and HSM tablets are mainly (more than 90%) made up of lactose and microcrystalline cellulose tableting materials, whose average particle sizes are 150 and 130 µm respectively.

**Figure 5:**
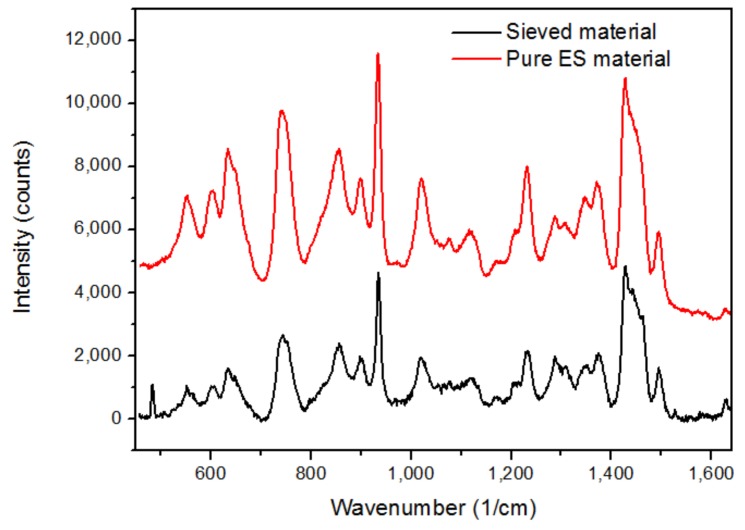
Raman spectra pure ES (red) and sieved (black) material.

According to the weighed sieve fractions, HSM homogenization was able to decrease the 355 µm portion by more than ten times compared to LSM mixing. This is crucial since the bulk of the pure electrospun materials was recovered on this sieve and, more importantly for complete homogenization, particle sizes should be as close to each other as possible to avoid segregation [55]. Based on these results, the high-performance impeller and chopper blades present in the HSM can further reduce the average particle sizes (due to high shear forces) of the fibrous materials, thus superior mixing of materials can be realized. These aforementioned fractions were also evaluated by Raman spectroscopy to look for traces of electrospun material. The spectra are shown in Figure 5.

It is clear that the spectrum of the sieved material is almost identical to the spectrum of the starting ES material ultimately proving that the API containing polymer was detected in the sieved mixture. In the end, this resulted in higher inhomogeneity among LSM tablets compared to HSM tablets. This outcome can be linked to Table 4 where the high SD and RSD values of the LSM tablets validate these findings.

Based on these results it is clear that a greater sample size was needed to be evaluated in order to identify the causes of homogeneity differences observed between the mixing methods. By implementing sieve analysis, the equivalent of 250 tablets could be screened, which enables broader examination compared to single tablet Raman mapping. 

This outcome proves that the high-shear mixer coupled with the direct compression technology should be the first method of choice when proper homogenization of electrospun samples is required.

## 4. Conclusions

A low-dose, highly homogenous tablet formulation made from drug-loaded electrospun fibers was demonstrated by applying HSM homogenization. Carvedilol, an antihypertensive drug was used as the model API, while PVPVA64 was adopted as the matrix forming polymer. Electrospinning was carried out by HSES with increased productivity, while the SNES technology was applied as the reference. Homogenizations were made in plastic bags (LSM) and in an HSM, while tableting was performed by a tablet rotary press. Tablet batches containing crystalline carvedilol served as reference. Sieve analysis was used to study the particle size distribution in the final LSM and HSM powder mixtures. API content and homogeneity were measured by UV-Vis spectrometry, while the latter was also studied by Raman mapping. 

HSM homogenization seems to be the preferred method of choice for preparing highly homogenous tablets containing electrospun NASDs. Their RSD values were much lower than their LSM counterparts. It is encouraging that this was achievable, despite setting a low API dose (50 µg) for the tablets. The technology can be scaled up easily, which could be truly beneficial for both academic researchers at universities and pharmaceutical technologists in the industry.

## Figures and Tables

**Table 1:** Tablet composition.

Applied Tableting Ingredients	Amount (mg)/Tablet	Amount (%)/Tablet	Total Amount of Materials (Batch Size 5000 Tablets)
Electrospun/Crys. Carvedilol	0.05	0.05	0.25 g
poly (vinylpyrrolidone-*co*-vinyl acetate) (PVPVA64)	4.95	4.95	24.75 g
Flowlac 100 mesh (amorphous lactose)	65.00	65.00	325 g
Microcrystalline cellulose (MCC) 112	25.50	25.50	127.5 g
Croscarmellose sodium (CCS)	3.00	3.00	15.0 g
Sodium stearyl fumarate (Pruv)	1.5	1.50	7.5 g
∑	100.0	100.0	500.00 g

**Table 2:** Comparison of the details of manufacturing using SNES and HSES.

Sample	Preparation Method	Applied Solvent	Dissolved PVPVA64 and CAR (99:1) in 10 mL of Solvent (g)	Flow Rate (mL/h)	Productivity for Dried Material (g/h)
PVPVA64 +1% CAR SNES	Single-needle electrospinning	96% EtOH	4.00	6	1.8
PVPVA64 +1% CAR HSES	High-speed electrospinning	96% EtOH	4.00	750	225

**Table 3:** Batch characteristics and their IPC results (A and B are repetitions of the same technology).

Characteristics	LSM Reference	HSM Reference	LSM HSES Batch A	LSM HSES Batch B	HSM HSES Batch A	HSM HSES Batch B
API type	Crystalline CAR	Crystalline CAR	Electrospun CAR	Electrospun CAR	Electrospun CAR	Electrospun CAR
Method of homogenization	Manual (LSM)	High-shear (HSM)	Manual (LSM)	Manual (LSM)	High-shear (HSM)	High-shear (HSM)
Weight (mg)	99.9 ± 0.5	99.2 ± 0.7	99.2 ± 0.8	100.2 ± 0.7	100.9 ± 1.0	99.8 ± 1.1
Thickness (mm)	3.08 ± 0.44	3.02 ± 0.54	3.06 ± 0.69	3.04 ± 0.57	3.05 ± 0.60	3.03 ± 0.52
Hardness (N)	64.3 ± 7.62	69.2 ± 6.22	60.6 ± 5.67	63.8 ± 7.51	68.6 ± 5.11	62.7 ± 6.71
Diameter (mm)	5.97 ± 0.19	5.95 ± 0.22	5.97 ± 0.27	5.98 ± 0.17	5.99 ± 0.29	5.97 ± 0.21
Friability (%)	0.21	0.31	0.39	0.25	0.20	0.27
Disintegration (s)	84	95	103	119	138	125

**Table 4:** UV-Vis results.

Sample Number	Carvedilol %					
	LSM Reference	HSM Reference	LSM ES A	LSM ES B	HSM ES A	HSM ES B
Average	98.76	97.68	94.19	93.21	97.63	96.71
SD (%)	7.43	4.65	9.50	6.80	2.04	2.49
RSD	7.52	5.13	10.08	7.30	2.09	2.58

**Table 5:** Sieve analysis results (the mixtures contain 5% ES material).

Sieve Size (µm)	Pure ES Material (g)	LSM Final Powder Mixture (g)	HSM Final Powder Mixture (g)
355	13.80	0.22	0.02
180	1.61	5.02	5.41
90	1.03	9.22	9.35
sub 90	8.56	10.54	10.22
